# A qualitative study of the impact of a dementia experiential learning project on pre-medical students: a friend for Rachel

**DOI:** 10.1186/s12909-019-1565-3

**Published:** 2019-05-02

**Authors:** Jill S. Goldman, Amy E. Trommer

**Affiliations:** 1 Taub Institute for Research on Alzheimer’s Disease and the Aging Brain, Columbia University Medical Center, 630 W. 168th St., Box 16, New York, NY 10032 USA; 2 Eldercare Innovations, LLC, 8 Little John Place, White Plains, NY 10605 USA

**Keywords:** Experiential learning, Dementia, Alzheimer’s disease, Pre-medical education, Service learning

## Abstract

**Background:**

With Alzheimer’s disease and other dementias affecting approximately 7 million people in the United States, comprehension of the multitude of issues facing individuals with dementia and their families and compassion for them are essential components of good healthcare. The service learning program, *A Friend for Rachel*, was developed in 2011 to train pre-medical students about dementia and give them sustained exposure to people with dementia to foster understanding and compassion and decrease stigma,. The purpose of this study was to evaluate the impact of the program on pre-medical students.

**Methods:**

Since 2011, 101 students participated in *A Friend for Rachel*. They were required to write weekly reflections about their interactions with their friends living with dementia. Each study author read these reflections to identify major recurrent themes. The authors discussed the themes and came to consensus. The reflections were then reread to analyze for sub-themes.

**Results:**

Analysis of students’ reflections exposed five major themes: learnings about dementia, learnings about caregiving, their own experienced emotions, impact on career choice and learnings about good medicine, and impact on life. The reflections demonstrated appreciation of the issues raised by dementia, empathy for individuals living with dementia and their families, and comfort with people with dementia. The reflections also demonstrated how the program had a positive impact on the personal lives of the students.

**Conclusions:**

Through experiencing a sustained relationship with a person living with dementia, *A Friend for Rachel* allows pre-medical students to re-evaluate their beliefs about dementia and appreciate the need for compassionate care for people with dementia. *A Friend for Rachel* also provides students with the opportunity to examine their personal lives and goals.

## Background

In the United States, 5.7 million people are living with Alzheimer’s disease (AD) [1]. The prevalence of all dementias in the U.S. is estimated to be about 7 million [1]. The actual monetary cost and impact on the health and well-being of caregivers is staggering [1]. Enhancement of future dementia care is dependent on developing a healthcare system that creates a better quality of life for both those with dementia and their caregivers. An appreciation of these needs should begin early in the careers of medical professionals.

Giving a diagnosis of dementia is a thankless task for any physician, but one that demands understanding and empathy. Frequently, the patient is seen as the sum of his symptoms rather than a whole person [2]. This view diminishes the patient’s sense of self and purpose, potentially making him less able to cope [3] and even reducing performance on clinical cognitive testing [4].

Both attitude toward and lack of knowledge about dementia potentially may influence how medical students and physicians treat their patients. In one study, only 2.5% of first year medical students and 68.0% of fourth year students had a good knowledge of AD. Personal experience with dementia resulted in more knowledge [5]. Attitudes about dementia may pre-date actual clinical experience [6–9]. A number of different educational interventions have been designed to improve knowledge and attitudes. The great majority demonstrate a positive outcome which is improved further with an experiential learning component [8, 10–13]. However, service learning without formal training can induce negative reactions [14].

One could infer from these studies that medical education about dementia could be improved by extensive direct contact with people with dementia. However, the demands of medical education do not allow for long-term one-on-one experiences. Thus, *A Friend for Rachel* (AFFR) was designed as an experiential learning project for undergraduate and post-baccalaureate students preparing for medical school (henceforth referred to as pre-medical students).

Measuring outcomes of a program such as AFFR is difficult. Feedback from family caregivers can provide information on their loved ones’ mood after outings, but long-term change is not expected because of the progressive and highly fluctuating nature of the diseases. Measuring change in students’ knowledge and attitudes is achievable; however, after several years of administering before/after questionnaires about knowledge and attitudes (e.g. Alzheimer’s Disease Knowledge Scale [15], Dementia Attitude Scale [16]) and finding the expected results of increased knowledge, reduced stigma and fear of dementia, and greater acceptance of people with dementia, we felt that these measures did not reflect the greater experiential learning achieved by the experience [17]. Thus, a qualitative study of the weekly student reflections was initiated in order to determine how their experiences in AFFR affected their thoughts about dementia.

## Methods

### Program description

Since its onset in 2011, 101 students have participated in AFFR. These students made a commitment of at least a full academic year, which includes eight training workshops through the year, weekly 3–4 h meetings with their “friends”- people with early to moderate stage dementia, and emailed weekly reflections shared with the coordinators for supervision. The goals of the program are three-fold: 1. For people with dementia: improved quality of life by continuing activities they enjoy both in and especially out of the home; decreased isolation; a meaningful relationship with a student friend to share their life history, knowledge, feelings, and to mentor. 2. For the family caregivers: respite time and support. 3. For the student friends: realistic perspective on dementia and reduced stigma; knowledge and awareness of the complexities of living with dementia; appreciation of the whole person. The program was developed and facilitated by this paper’s authors.

### Participants and recruitment

Upon entering the program, the majority of participants in AFFR were juniors, seniors, and post-baccalaureate students in the pre-medical track at Columbia University and Barnard College. A few participants were sophomores or had already graduated from the University and were in their gap year before pursuing medical school or another graduate health program. Approximately 35 % of the participants remained in the program for 2–3 years, and many of these had graduated by the time they completed their AFFR participation*.*

Participants were recruited with help from the Columbia office for pre-professional advising through a campus-wide “E-blast” with the application announcing the program. An additional announcement was made through the post-baccalaureate program newsletter. Other students were referred by past participants. Students were invited to attend an information session about the program. They then completed an application and an in-person interview.

### AFFR requirements

Students accepted into the program committed to being paired with a person with early to moderate dementia with whom they visited for 3–4 h weekly. Additional requirements included weekly emailed reflections about their visits and attendance at 4 training workshops per semester. Students were asked to include descriptions of the activities they engaged in with their friends, their perceptions about the outings with their friends, their friends’ reactions to these activities, and any learnings resulting from the encounter. The coordinators reiterated this requirement throughout the program. Yearly trainings included a discussion with a neurologist about dementia diagnosis and management, psychological aspects of dementia, caregiving issues, communication techniques to use with people with dementia and how to model these techniques for the caregivers, how to best manage “difficult” behaviors, activities for people with dementia utilizing art, music, and dance, a panel of people with early-stage dementia, and a caregiver panel. All sessions included time for sharing, peer support, and guidance from the study authors.

### Informed consent

All students participating in *A Friend for Rachel* were invited to participate in this study. All students agreed to participate and signed an informed consent approved by the Columbia University Medical Center Internal Review Board.

### Consent to publish

Not applicable.

### Study design and analysis

Students’ weekly reflections from the 7 years of the program were printed and read by each study author to identify major recurrent themes. Approximately 1200 emailed reflections from 95 students were included in the analysis. Reflections from our first year of the program were unfortunately lost. The reflections included thoughts on the students’ weekly visits with their friends as well as on the trainings. The inductive data driven approach was used to analyze the phenomenology of the student experience [18]. Each author read the reflections looking for repeated ideas and topics and recorded these. Each author then grouped similar themes into major themes. The authors discussed the themes and came to consensus. The reflections were then re-read by both authors to analyze for sub-themes. With discussion and rereading, these subthemes were culled to those most frequently mentioned within each theme [19]. Reflections were then reread to derive supporting quotes for each sub-theme as well as quotes that were particularly meaningful for each theme.

## Results

Student reflections on their encounters and their trainings were classified into five major themes. Sub- themes and representative comments are presented in Table 1. Additional findings and a few longer substantive quotes are presented below.

**Table 1:** Student reflections’ themes and subthemes

Themes and subthemes	Reflection illustrating subthemes
*Theme 1: Learnings about dementia and aging*	
• Appreciation of person’s life experience and knowledge.	“He may not remember present details, but his memory of poetry and music is astounding.”
• Ability to still enjoy life, resiliency.	*“*She was an artist. All around her apartment was her artwork—evidence that she was a living, breathing, and experiencing individual. It was amazing to think that despite the toll that Alzheimer’s was taking on her word-retrieval abilities and other aspects of her memory, her ability to imagine and create beautiful images on a canvas was left intact.”
• Challenging behaviors	“It’s fascinating (and heartbreaking) that there can be such dramatic ups and downs.”
• Need for social interaction.	“She is one of the friendliest people I have met,. .. based on a few bad experiences with some people since she found out about her dementia, she’s become less sure of gatherings with strangers. One of her closest friends no longer talks to her and that has made a significant impact on her.”
• Need for control and validation, stigma	“…it makes her feel like her disease makes people think she is completely incapable of living independently. She understands that she does forget things, but that doesn’t mean that she is completely incapacitated. These incidents have been frustrating her because she currently still feels capable of doing a lot of things”
*Theme 2: Learnings about caregiving*	
• Isolation, burden	“I recall the exhaustion and sense of exasperation in his wife’s answers. X, however, seemed to be cheery with a happy go lucky attitude. This contrast particularly struck me because it can be easy to just focus solely on the person with dementia, but it’s just as important to the person’s well-being that the people supporting them are considered too.”
• Coping mechanisms	“He would be overly cautious with her and it makes her feel more incapable than she feels.”.
• Need for education, support, validation	“She mentioned that he has been going to caregiver-only support groups and how that has aggravated the situation since the other caregivers in the group have loved ones that are further along in the disease. She said that it has made him more paranoid about her situation and she found that he comes back from the meetings unhappy/worried.”
*Theme 3: Emotions experienced*	
• Frustration, disappointment, anxiety	“It helped me understand how frustrated family members and friends of people with dementia can get, Overcoming that frustration was a very important learning experience.”
• Sadness	“He continued by saying something felt wrong that he had to figure out. Hearing him say this filled me sadness.”
• Satisfaction and joy	“He then shared how much he enjoyed spending time with me. This made me feel appreciated. I remember thinking and hoping that he knew how much I appreciated spending time with him.”
• Acceptance	“Even when she isn’t making sense, she is still this lovely, funny, happy person who is a joy to be around.”
*Theme 4: Impact on career choice and learning about good medicine*	
• Reinforced desire to become a doctor	“He reminded me why I am pursuing medicine-to be able to touch the life of another person in a tangible, meaningful way. But medicine is not a one-way street-…practicing medicine also means allowing the other person to touch you…”
• Doctors must validate strengths rather than weaknesses.	“Only by encouraging a grateful attitude and emphasizing life rather than death can we, as future physicians, ensure the best quality of life for individuals with dementia.”
• Doctors must be good listeners, be tactful, and be sensitive	“…medicine is truly the art of human connections… being sensitive to my patient means being aware of their emotional reactions and incorporating their experience into the type of care I will give”.
*Theme 5: Impact on life*	
• Importance of relationships	“I don’t think I realized how much our relationship benefits both of us. While I may provide some youthful energy, he imparts his priceless wisdom and steadfast encouragement.”
• Importance of being in the moment	“Humor is ultimately another way of making life in the present more livable. Yes, life doesn’t always work out the way you want it to. But being happy in the moment and cherishing the good days is the only way to live.”
• Spend more time with family and friends	“Spending time with him has given me more confidence in my ability to interact with patients, it has also made me appreciate the relationships I have with my family.”

### Theme 1: learnings about dementia

Student reflections demonstrated shifting attitudes about dementia and appreciation for their friends. Through interactions between their friends and other people, they observed the hurtful stigma and misconceptions about dementia. Many admitted to originally thinking that all dementia was the same. They had not considered disease progression, different types of dementia, or innate differences in people with dementia.

Students witnessed the struggles that accompany dementia. They discussed watching fluctuations in cognition and behavior and increased anxiety associated with confusion. Many experienced sadness watching their friends’ physical and cognitive decline and the associated frustration. They noted that their friends often did or said inappropriate things, resulting in embarrassing situations. When seeing old food in the refrigerator or recognizing a missed meal, they feared for those living alone who refused increased assistance. Reflections discussed the friends’ isolation and limited social interactions. Some of the senior friends spoke of being a burden to loved ones, and expressed appreciation for the time they spent with their student friends who were non-judgmental and appreciative of their present selves. Students spoke about control issues between spouses, and how much their friends disliked being treated like children. The students, too, often struggled with how to balance their friends’ safety and their need for independence, and found ingenious ways to validate them and allow them a sense of control.

The great majority of reflections, however, expressed positive attitudes about dementia. Students discussed how much they learned from their friends and how they often relied on them for insights about their own lives. They noted that their friends gained a renewed sense of purpose and enjoyment by being mentors. They commented on their friends’ ability to look at the world with childlike wonder and enjoy being in the moment. Students realized how their friends retained emotional memories over factual ones. They acknowledged that their friends often forgot what they had done together, but recalled enjoying the time they had spent. They saw how art and music evoked positive emotions while the need to remember facts led to agitation. Several mentioned that their friends spoke of the grief they felt upon diagnosis and at the loss of their abilities, but also of the determination to enjoy life.

### Theme 2: learnings about caregiving

The most common reflections highlighted the physical and emotional burden experienced by the caregivers. Students commented on the required time and energy, the frustration and anger, the isolation, and the need for tremendous patience and flexibility. Students recognized that the most effective caregivers were flexible. Maintaining a rigid schedule/attitude often resulted in frustration for both the caregiver and person with dementia. Students learned to be flexible about their own plans and be “in the moment.”

Some noted situations where families came together and shared caregiving, others where family members did not participate. They realized how much dementia affects the whole family. They noted how reliant the caregiver was on them for respite time and support, and how honored they were to act as a confidante.

Many students reflected on how caregivers needed more support and validation, and how they often felt unappreciated by the person with dementia, by their families, and by society in general. They observed ineffectual caregiving and struggled with how to intercede. When listening to a panel of caregivers and to a panel of early-stage dementia patients, students reflected on the difference in perspectives: *“It made me realize again the crucial importance of listening to ‘caretakers’ voices and their narratives. They are such a vital part in this whole dialogue of providing medical care. As an aspiring physician, I want to be able to better incorporate their experiences into the empathetic care I hope to give. An illness does not only affect the patient; an illness affects everyone surrounding that person. And thus, it’s necessary for doctors to recognize this and use it to better treat and care for patients.”*

Students noted caregivers’ positive and negative coping mechanisms. They talked about denial, over-control, humor, love, and grief. Students watched the caregivers’ slow acceptance of loss. Some students saw caregivers find a new purpose in life and a new appreciation of their marriage. They noted resilience and courage.

### Theme 3: emotions experienced

All students experienced a wide spectrum of emotions. Most students entered their relationships with enthusiasm but trepidation and anxiety. For fear of not knowing what to do or say, they pre-planned their outings, sometimes discovering that their plans did not happen the way they anticipated. Some students expressed disappointment, frustration, and a sense of failure. They often identified with the family caregivers.

One student acknowledging her frustration said: *“It takes her longer to find words, and she garbles words more often. I am trying not to get frustrated with myself when it takes me a moment to understand what she is saying, because I really want her to feel comfortable with me and not like she is working extra hard to communicate, but sometimes it’s still hard. I’m also finding that she is having a difficult time grasping overall concepts in a way that surprises me, given her intelligence.”*

When watching their friends’ dementia progress, the students experienced sadness and loss. Several needed to be “re-matched” because their friend’s progression was faster than predicted and could no longer participate in the program. One student wrote

*“I've been thinking more about making moments and days and conversations count with people we love, because everything can clearly become altered in an instant.”*

Students also experienced significant positive emotions. They felt privileged that they could provide support to caregivers and felt immense satisfaction at impacting their friends’ lives, and happiness when they saw their friends’ joy. Over time, students became more flexible and comfortable with living in the moment. Some learned to accept the limits of communication, and became more comfortable with silence and non-verbal communication. They accepted their friends’ fluctuations, and eventual loss. Some commented that they feared aging less than before knowing their friend.

### Theme 4: impact on career choice and learnings about good medicine

A significant number of the students applying for AFFR had an interest in neuroscience or a relative with dementia. However, few had determined their exact career path or specialty. AFFR has led some students to consider geriatrics, end-of-life care, and neurology. Several students shifted their goal of an MD/PhD for research to an MD for patient care. Most students commented on how their experience reinforced their desire to become a doctor in order to help people. Many received encouragement from their senior friends, and thus felt more self-confident about their ability to interact and communicate with patients and their families.

Not only did students shore up their desire to become doctors, but also developed an appreciation of traits needed to be a good doctor. Reflections included comments senior friends and caregivers had made about their experiences with doctors. Students recognized that these negative interactions were due to insensitivity and inadequate listening, and felt that their own listening and communication skills had improved through AFFR.

After listening to a caregiver panel, two students reflected:

*“I thought a lot about how I hope to act as a doctor. I was astonished by how poor the patient-doctor interactions were according to these caregivers, especially with such sensitive, life-altering issues … I can only hope that as a physician, I will be able to display more compassion than what these caregivers experienced by remembering what I learned in this session. No one should ever be treated with indifference or coldness in the wake of devastating news, and I hope to make it my mission to change the face of health care in that regard in whatever capacity I can.”*

*“Maybe I’m naïve, but I would hope that physicians in particular would strive to keep their humanity. Physicians interact with human beings at their lowest points of life on an emotional and physical scale, so to lose humanity in this profession is a frightening thought”.*

### Theme 5: impact on life

Despite the reason for applying to *A Friend for Rachel*, few students expected to be changed by the experience. One student observed, *“It (the program) has affected the way I interact with people- peers, colleagues, strangers, and family members. Oftentimes, when interacting with others, I am self-focused and caring a lot about how others see me and what I should get done with the other person. When I spend time with my friend with dementia, I find that this way of thinking is not at all effective. Not only does it make me anxious, but it also prevents a real connection forming between the two of us. Only when I started letting my own plans go and started to go with the flow could I really listen to what she had to share. When I started to live in the moment, I began to truly enjoy my time with my friend…Now I value all my friendships more and feel more comfortable getting to know each other.”*

Students realized how their friends taught them about the importance of gratefulness, having a positive outlook, and having meaningful and supportive relationships. They developed a greater appreciation of all their relationships, and felt that by listening more effectively, they were becoming better friends. They saw themselves looking at strengths rather than weaknesses. Students observed how their senior friends responded to positive statements and encouragement and generalized this to people in their broader life. They noted how they had learned not to correct, but instead to listen and validate.

Students learned to appreciate being in the moment and to look at things differently. Numerous students talked about noticing nature and even the architecture of building. They came to realize that they were so self- and future-focused that they forget to enjoy the present. Despite being in New York for 3–4 years, some students had barely left campus until they went exploring with their senior friend.

Being with their friends made students more aware of mortality and the importance of prioritizing time for meaningful relationships. They thought more about their families, especially grandparents, and wanted to spend more quality time with them. Many students thought about the relatives who were lost to dementia, and regretted not spending more time with them. Several students came to redefine friendship, and realized the necessity of sharing their own thoughts and emotions.

## Discussion

The weekly narratives of their experiences and thoughts demonstrate the impact of AFFR on the pre-medical students. Narrative medicine has been shown to be an effective way to enhance learning and is being incorporated into medical school curricula [20]. Through journaling, students take time to process their thoughts and experiences and reflect on how they see themselves changing. The AFFR reflections demonstrated attitudinal change and self-learning. The reflections revealed decreased stigma and fear of dementia and improved empathy, flexibility, appreciation of living in the moment, and communication skills (both verbal and nonverbal). Students learned the importance of treating people with dignity and appreciation. They reflected on the lives of people with dementia and their caregivers, but also on their own lives including: their shortcomings, changes in themselves, and their priorities and goals. They applied what they learned from their interaction with people with dementia to their everyday lives.

When applying to AFFR, few students considered how the program would influence their overall lives. They thought about helping people with dementia, learning about clinical aspects of dementia, having a hands-on experience with patients, and about medical school recommendations. Some had grandparents with dementia and had enrolled in AFFR with the hope of helping their parents. Some had parents who had been recently diagnosed, and they felt the need to understand what the future might bring*.* For some, the program was a way to improve communication skills, challenge themselves, or push out of their comfort zone. Surprisingly over time, however, they discovered that AFFR had a far greater impact.

As stated in the introduction, many students enter medical school with a lack of knowledge and negative attitudes about dementia. Various programs have been designed to counter these issues. The majority of those that have had positive outcomes have incorporated experiential learning with formal training. *A Friend for Rachel*, incorporating experiential learning, training, and mentorship, was designed to provide students with a meaningful, prolonged relationship with a person with dementia. AFFR demonstrates that attitudes about dementia can change significantly after a long-term relationship with a person living with dementia. Past research on dementia service learning projects have shown conflicting results [21–24]. Many of these studies were on classroom–based programs with additional time spent in long-term care facilities. *A Friend for Rachel*’s design was significantly different in several ways. The student training sessions were experiential, not lecture-based. Trainings included panels of people with dementia and their caregivers, discussions with neurologists, improvisation and communication workshops, and discussions about both positive and negative experiences with their friends. Whereas in past service learning programs, the great majority of student/people with dementia interactions took place in long-term care facilities or a fixed environment with people who were in mid- to late-stage disease, the participants in AFFR were mildly or moderately affected, and the goal of interactions was to enjoy activities both in the home and around the city. Additionally, students in AFFR committed to seeing their friend for 3–4 h a week for at least an entire academic year, which was a significantly longer time than in most other programs. By doing so, they built a strong relationship with their friend and developed an understanding of the caregiver. Lastly, students were supervised on a weekly basis by the project coordinators (authors) who are specialists in the dementia field. Such mentoring has been shown to facilitate attitudinal change [25]. One successful service learning program, Opening Minds through Art, paired a student with a person with mild dementia for 10–12 weeks in which they participated together in art projects. Like our program, students received training in dementia, person-centered care principles, and communication skills. The outcomes included not only a decrease in negative attitudes toward people with dementia but an increase in positive attitudes [9]. However, the program differed from AFFR by its fixed activity, site, and shorter duration. Studies indicate a positive correlation between students’ attitudes and the number of positive interactions they have with people with dementia [26].

Limitations to this study include the selection bias of the student participants. The AFFR students volunteer for the program and may already have better attitudes about dementia than the general student population. However, even within this group, we could register change.

## Conclusions

Through weekly one-on-one interactions with people with dementia and their families, on-going trainings, and supervision, *A Friend for Rachel* allowed pre-medical students to re-evaluate their beliefs about dementia and appreciate the need for compassionate care and an individualized approach for people with dementia. Students became more empathetic, less controlling, and more appreciative of living in the moment. *A Friend for Rachel* also provided students with the opportunity to examine their personal lives and goals. For most participants, *A Friend for Rachel* strengthened their commitment to become physicians. They learned essential professional skills, including: patient centered care, communication skills, empathy, observational skills, quality of care, importance of family involvement, and impact of disease on families. They recognized the need for physicians not only to provide good healthcare, but also understanding and empathy for their patients and families whether living with dementia or other conditions.

## Abbreviations

AD: Alzheimer disease
AFFR: A Friend for Rachel

## References

[1] Alzheimer’s Association (2018). Alzheimer’s Disease Facts and Figures.

[2] Kitwood T (1997). Dementia reconsidered: the person comes first.

[3] La Fontaine J, Oyebode JR (2014). Family relationships and dementia: a synthesis of qualitative research including the person with dementia. Aging Soc.

[4] Levy B (1996). Improving memory in old age through implicit self-stereotyping. J Pers Soc Psychol.

[5] Nagle BJ, Usita PM, Edland SD (2013). United States medical students’ knowledge of Alzheimer disease. J Educ Eval Health Prof.

[6] Reuben DB, Fullerton JT, Tschann JM, Croughan-Minihane M (1995). Attitudes of beginning medical students toward older persons: a five-campus study. The University of California Academic Geriatric Resource Program Student Survey Research Group. J Am Geriatr Soc.

[7] Fitzgerald JT, Wray LA, Halter JB, Williams BC, Supiano MA (2003). Relating medical students’ knowledge, attitudes, and experience to an interest in geriatric medicine. Gerontologist..

[8] Jefferson AL, Cantwell NG, Byerly LK, Morhardt D (2012). Medical student education program in Alzheimer’s disease: The PAIRS Program. BMC Med Educ.

[9] Lokon E, Kunkel S (2018). Allophilia: increasing college students’ “liking” of older adults with dementia through arts-based intergenerational experiences. Gerontol Geriatr Educ.

[10] Wood JH, Alushi L, Hammond JA (2017). Communication and respect for people with dementia: student learning - a novel practical experience of undergraduate students interacting with people with dementia in care homes (innovative practice). Dementia (London).

[11] Shue CK, McNeley K, Arnold L (2005). Changing medical students’ attitudes about older adults and future older patients. Acad Med.

[12] Kalisch HR, Coughlin DR, Ballard SM, Lamson A (2013). Old age is a part of living: student reflections on intergenerational service-learning. Gerontol Geriatr Educ..

[13] Brown KM, Bright LM (2017). Teaching caring and competence: student transformation during an older adult focused service-learning course. Nurse Educ Pract.

[14] Robinson A, Cubit K (2007). Caring for older people with dementia in residential care: nursing students’ experiences. J Adv Nurs.

[15] Carpenter B, Balsis S, Otilingam PG, Hanson PK, Gatz M (2009). The Alzheimer’s disease knowledge scale: development and psychometric properties. Gerontologist..

[16] O’Connor M, McFadden S. Development and psychometric validation of dementia attitude score. Int J Alzheimers Dis. 2010;2010:1–10.

[17] Goldman JS, Trommer A (2014). A friend for Rachel: an experiential learning project. Alzheimer's Dement.

[18] Hanson JL, Balmer DF, Giardino AP (2011). Qualitative research methods for medical educators. Acad Pediatr.

[19] Creswell JW (2003). Qualitative procedures. Research design: qualitative, quantitative, and mixed methods approaches.

[20] Cunningham H, Taylor D, Desai UA, Quiah SC, Kaplan B, Fei L, Catallozzi M, Richards B, Balmer DF, Charon R (2018). Looking Back to move forward: first-year medical Students’ meta-reflections on their narrative portfolio writings. Acad Med.

[21] Blieszner R, Artale LM (2001). Benefits of intergenerational service-learning to human services majors. Educ Gerontol.

[22] Fruhauf CA, Jarrott SE, Lambert-Shute J (2004). Service-learners at dementia care programs: An intervention for improving contact, comfort, and attitudes. Gerontol Geriatr Educ.

[23] Lambert-Shute J, Jarrott SE, Fruhauf CA (2004). Service-learning at dementia care programs: An orientation and training program. Gerontol Geriatr Educ.

[24] Yamashita T, Kinney JM, Lokon EJ (2013). The impact of a gerontology course and a service-learning program on college students’ attitudes toward people with dementia. J Appl Gerontol.

[25] Shield RR, Farrell TW, Campbell SE, Nanda A, Wetle T (2015). Professional development and exposure to geriatrics: medical student perspectives from narrative journals. Gerontol Geriatr Educ..

[26] Maharaj T (2017). Live-model simulation: improving nursing students’ attitudes and knowledge of Alzheimer’s disease. Clin Simul Nurs.

